# Deep neural networks for accurate predictions of crystal stability

**DOI:** 10.1038/s41467-018-06322-x

**Published:** 2018-09-18

**Authors:** Weike Ye, Chi Chen, Zhenbin Wang, Iek-Heng Chu, Shyue Ping Ong

**Affiliations:** 1 Department of Chemistry and Biochemistry, University of California San Diego, 9500 Gilman Dr, Mail Code 0303, La Jolla, CA 92093-0448 USA; 2 Department of NanoEngineering, University of California San Diego, 9500 Gilman Dr, Mail Code, 0448, La Jolla, CA 92093-0448 USA

## Abstract

Predicting the stability of crystals is one of the central problems in materials science. Today, density functional theory (DFT) calculations remain comparatively expensive and scale poorly with system size. Here we show that deep neural networks utilizing just two descriptors—the Pauling electronegativity and ionic radii—can predict the DFT formation energies of C_3_A_2_D_3_O_12_ garnets and ABO_3_ perovskites with low mean absolute errors (MAEs) of 7–10 meV atom^−1^ and 20–34 meV atom^−1^, respectively, well within the limits of DFT accuracy. Further extension to mixed garnets and perovskites with little loss in accuracy can be achieved using a binary encoding scheme, addressing a critical gap in the extension of machine-learning models from fixed stoichiometry crystals to infinite universe of mixed-species crystals. Finally, we demonstrate the potential of these models to rapidly transverse vast chemical spaces to accurately identify stable compositions, accelerating the discovery of novel materials with potentially superior properties.

## Abstract (web summary)

Crystal stability prediction is of paramount importance for novel material discovery, with theoretical approaches alternative to expensive standard schemes highly desired. Here the authors develop a deep learning approach which, just using two descriptors, provides crystalline formation energies with very high accuracy.

## Introduction

The formation energy of a crystal is a key metric of its stability and synthesizability. It is typically defined relative to constituent unary/binary phases (*E*_*f*_) or the stable linear combination of competing phases in the phase diagram (*E*_hull_, or energy above convex hull)^1^. In recent years, machine learning (ML) models trained on density functional theory (DFT)^2^ calculations have garnered widespread interest as a means to scale quantitative predictions of materials properties^3–7^, including energies of crystals. However, most previous efforts at predicting *E*_*f*_ or *E*_hull_ of crystals^5,8–12^ using ML models have yielded mean absolute errors (MAEs) of 70–100 meV atom^−1^, falling far short of the necessary accuracy for useful crystal stability predictions. This is because approximately 90% of the crystals in the Inorganic Crystal Structure Database (ICSD) have *E*_hull_ < 70 meV atom^−1^^13^, and the errors of DFT-calculated formation energies of ternary oxides from binary oxides relative to experiments are ~ 24 meV atom^−1^^14^.

We propose to approach the crystal stability prediction problem by using artificial neural networks (ANNs)^15^, i.e., algorithms that are loosely modeled on the animal brain, to quantify well-established chemical intuition. The Pauling electronegativity and ionic radii guide much of our understanding about the bonding and stability of crystals today, for example, in the form of Pauling’s five rules^16^ and the Goldschmidt tolerance factor for perovskites^17^. Though these rules are qualitative in nature, their great success points to the potential existence of a direct relationship between crystal stability and these descriptors.

**Fig. 1:**
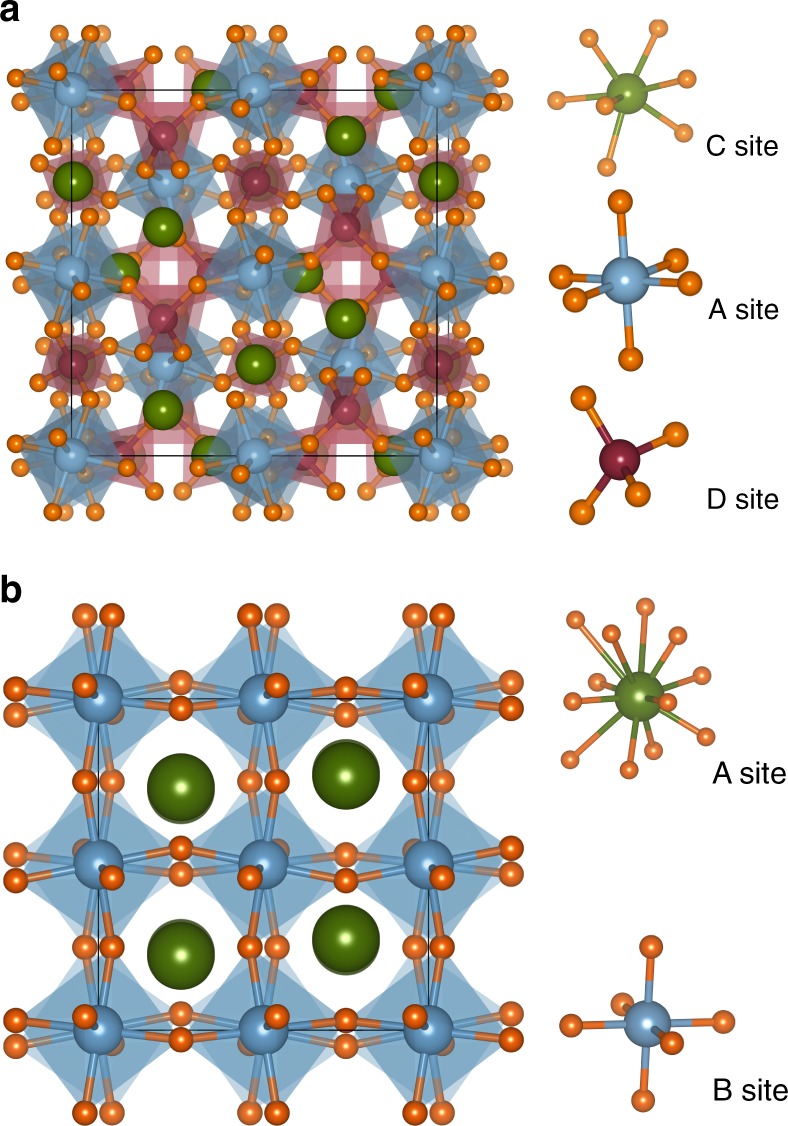
Crystal structures of garnet and perovskite prototypes. **a** Crystal structure of $Ia\overline 3 d$ C_3_A_2_D_3_O_12_ garnet prototype. Green (C), blue (A), and red (D) spheres are atoms in the 24*c* (dodecahedron), 16*a* (octahedron), and 24*d* (tetrahedron) sites, respectively. The orange spheres are oxygen atoms. **b** Crystal structure of *Pnma* ABO_3_ perovskite prototype. Green (A) and blue (B) spheres are atoms in the 4*c* (cuboctahedron) and 4*d* (octahedron) sites, respectively. The orange spheres are oxygen atoms

To probe these relationships, we choose, as our initial model system, the garnets, a large family of crystals with widespread technological applications such as luminescent materials for solid-state lighting^18^ and lithium superionic conductors for rechargeable lithium-ion batteries^19,20^. Garnets have the general formula C_3_A_2_D_3_O_12_, where C, A and D denote the three cation sites with Wyckoff symbols 24*c* (dodecahedron), 16*a* (octahedron) and 24*d* (tetrahedron), respectively, in the prototypical cubic $Ia\overline 3 d$ garnet crystal shown in Fig. 1a. The distinct coordination environments of the three sites result in different minimum ionic radii ratios (and hence, species preference) according to Pauling’s first rule. We further demonstrate the generalizability of our approach to the ABO_3_ perovskites (Fig. 1b), another broad class of technologically important crystals^21–25^.

In this work, we show that ANNs using only the Pauling electronegativity^26^ and ionic radii^27^ of the constituent species as the input descriptors can achieve extremely low MAEs of 7–10 meV atom^−1^ and 20–34 meV atom^−1^ in predicting the formation energies of garnets and perovskites, respectively. We also introduce two alternative approaches to extend such ANN models beyond simple unmixed crystals to the much larger universe of mixed cation crystals—a rigorously defined averaging scheme for the electronegativity and ionic radii for modeling complete cation disorder, and a novel binary encoding scheme to account for the effect of cation orderings with minimal increase in feature dimension. Finally, we demonstrate the application of the NN models in accurately and efficiently identifying stable compositions out of thousands of garnet and perovskite candidates, greatly expanding the space for the discovery of materials with potentially superior properties.

## Results

### Model construction and definitions

We start with the hypothesis that the formation energy *E*_*f*_ of a C_3_A_2_D_3_O_12_ garnet is some unknown function *f* of the Pauling electronegativities (*χ*) and Shannon ionic radii (*r*) of the species in the C, A, and D sites, i.e.,

1 $$E_f = f\left( {\chi _{\mathrm{C}},\,r_{\mathrm{C}},\chi _{\mathrm{A}},r_{\mathrm{A}},\,\chi _{\mathrm{D}},r_{\mathrm{D}}} \right)$$

Here, we define *E*_*f*_ as the change in energy in forming the garnet from binary oxides with elements in the same oxidation states, i.e., $E_f^{\mathrm{oxide}}$ as opposed to the more commonly used formation energy from the elements $E_f^{\mathrm{element}}$ in previous works^8–11^. Using the Ca_3_Al_2_Si_3_O_12_ garnet (grossular) as an example, $E_f^{\mathrm{oxide}}$ is given by the energy of the reaction: 3CaO + Al_2_O_3_ + 3SiO_2_ → Ca_3_Al_2_Si_3_O_12._ This choice of definition of *E*_*f*_ is motivated by two reasons. First, binary oxides are frequently used as synthesis precursors. Second, our definition ensures that garnets that share elements in the same oxidation states have *E*_*f*_ that are referenced to the same binary oxides, minimizing well-known DFT errors. In contrast, $E_f^{\mathrm{element}}$ and *E*_hull_ are both poor target metrics for a ML model. $E_f^{\mathrm{element}}$ suffers from non-systematic DFT errors associated with the incomplete cancellation of the self-interaction error in redox reactions^28^, while *E*_hull_ is defined with respect to the linear combination of stable phases at the C_3_A_2_D_3_O_12_ composition in the C-A-D-O phase diagram, which can vary unpredictably even for highly similar chemistries. Henceforth, the notation *E*_*f*_ in this work refers to $E_f^{\mathrm{oxide}}$ unless otherwise stated. The binary oxides used to calculate the *E*_*f*_ for garnets and perovskites are listed in Supplementary Table 1 and 2, respectively.

**Fig. 2:**
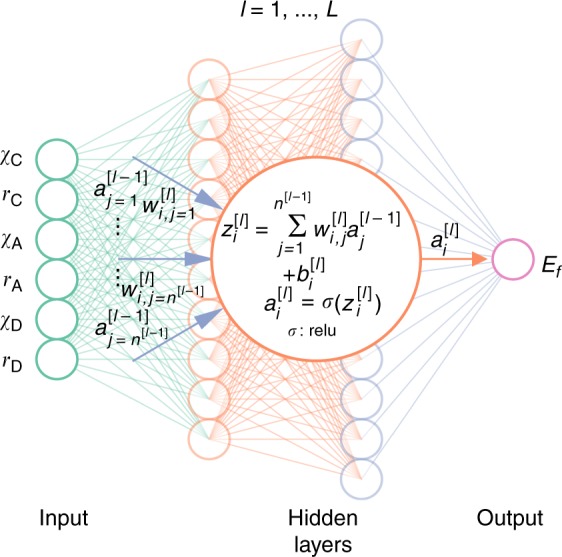
General schematic of the artificial neural network. The artificial neural network (ANN) comprises an input layer of descriptors (the Pauling electronegativity and ionic radii on each site), followed by a number of hidden layers, and finally an output layer (*E*_*f*_). The large circle in the centre shows how the output of the *i*_th_ neuron in *l*_th_ layer, $a_i^{\left[ l \right]}$, is related to the received inputs from (*l*−1)_th_ layer $a_j^{[l - 1]}$. $w_{i,j}^{\left[ l \right]}$ and $b_i^{\left[ l \right]}$ denote the weight and bias between the *j*_*t*h_ neuron in (*l*−1)_th_ layer and *i*_th_ neuron in *l*_th_ layer. *σ* is the activation function (rectified linear unit in this work). The ANN models were implemented using Keras^39^ deep learning library with the Tensorflow^40^ backend

Based on the universal approximation theorem^29^, we may model the unknown function f(*χ*_C_,*r*_C_,*χ*_A_,*r*_A_,*χ*_D_,*r*_D_), which is clearly non-linear (see Supplementary Fig. 1), using a feed-forward ANN, as depicted in Fig. 2. The loss function and evaluation metric are chosen to be the mean squared error (MSE) and MAE, respectively. We will denote the architecture of the ANN using *n*^*i*^−*n*^[1]^−*n*^[2]^−···−1, where *n*^*i*^ and *n*^[*l*]^ are the number of neurons in the input and *l*^th^ hidden layer, respectively.

### Neural network model for unmixed garnets

**Fig. 3:**
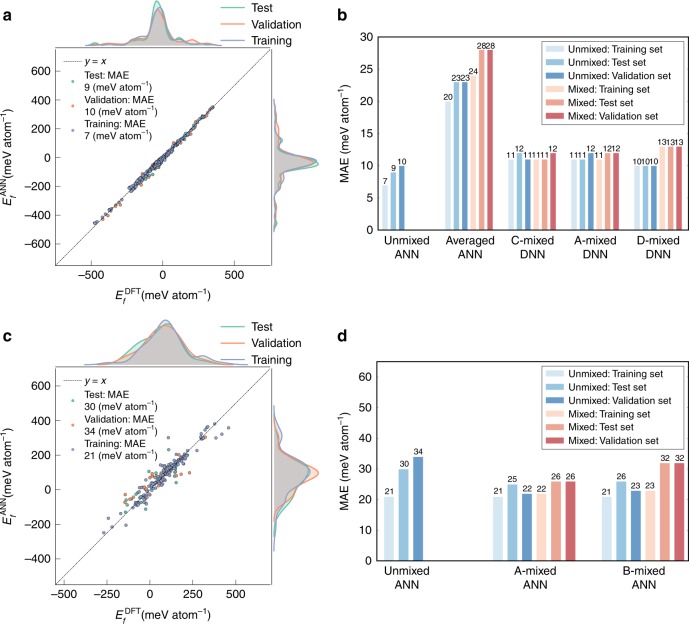
Performance of artificial neural network (ANN) models. **a** Plot of $E_f^{\mathrm{ANN}}$ against $E_f^{\mathrm{DFT}}$ of unmixed garnets for optimized 6-24-1 ANN model. The histograms at the top and right show that the training, validation and test sets contain a good spread of data across the entire energy range of interest with standard deviations of 122–134 meV atom^−1^. Low mean absolute errors (MAEs) in *E*_*f*_ of 7, 10, and 9 meV atom^−1^ are observed for the training, validation, and test sets, respectively. **b** MAEs in *E*_*f*_ of unmixed and mixed samples in training, validation, and test sets of all garnet models. The C-, A- and D-mixed deep neural networks (DNNs) have similar MAEs as the unmixed ANN model, indicating that the neural network has learned the effect of orderings on *E*_*f*_. Each C-, A- and D-mixed composition has 20, 18, and 7 distinct orderings, respectively, which are encoded using 5-bit, 5-bit, and 3-bit binary arrays, respectively. **c** MAEs in *E*_*f*_ of unmixed and mixed samples for training, validation and test sets of unmixed perovskites for 4-12-1 ANN model. The $E_f^{\mathrm{DFT}}$ of training, validation, and test sets similarly contain a good spread of data across the entire energy range of interest with standard deviations of 104–122 meV atom^−1^. Low mean absolute errors (MAEs) in *E*_*f*_ of 21, 34, and 30 meV atom^−1^ are observed for the training, validation, and test sets, respectively. **d** MAEs in *E*_*f*_ for training, validation, and test sets of all perovskite models. Each A- and B- mixed perovskite compositions has ten distinct orderings, which are both encoded using 4-bit binary arrays. The black lines (dashed) in (**a**, **c**) are the identity lines serving as references

We developed an initial ANN model for unmixed garnets, i.e., garnets with only one type of species each in C, A, and D. A data set comprising 635 unmixed garnets was generated by performing full DFT relaxation and energy calculations (see Methods) on all charge-neural combinations of allowed species (Supplementary Table 3) on the C, A, and D sites^30^. This dataset was randomly divided into training, validation, and test data in the ratio of 64:16:20. Using 50 repeated random sub-sampling cross validation, we find that a 6-24-1 ANN architecture yields a small root mean square error (RMSE) of 12 meV atom^−1^, as well as the smallest standard deviation in the RMSE among the 50 sub-samples (Supplementary Fig. 2a). The training, validation and test MAEs for the optimized 6-24-1 model are ~7–10 meV atom^−1^ (Fig. 3a), an order of magnitude lower than the ~100 meV atom^−1^ achieved in previous ML models^5,8–10^. For comparison, the error in the DFT *E*_*f*_ of garnets relative to experimental values is around 14 meV atom^−1^ (Supplementary Table 4). Similar RMSEs are obtained for deep neural network (DNN) architectures containing two hidden layers (Supplementary Fig. 2b), indicating that a single-hidden-layer architecture is sufficient to model the relationship *E*_*f*_ and the descriptors.

### Averaged neural network models for mixed garnets

To extend our model to mixed garnets, i.e., garnets with more than one type of species in the C, A, and D sites, we explored two alternative approaches—one based on averaging of descriptors, and another based on expanding the number of descriptors to account for the effect or species ordering. The data set for mixed garnets were created using the same species pool, but allowing two species to occupy one of the sites. Mixing on the A sites was set at a 1:1 ratio, and that on the C and D sites was set at a 2:1 ratio, generating garnets of the form C_3_A’A”D_3_O_12_ (211 compositions), C’C’’_2_A_2_D_3_O_12_ (445 compositions), and C_3_A_2_D’D’’_2_O_12_ (116 compositions)_._ For each composition, we calculated the energies of all symmetrically distinct orderings within a single primitive unit cell of the garnet. All orderings must belong to a subgroup of the $Ia\overline 3 d$ garnet space group.

In the first approach, we characterized each C, A, or D site using weighted averages of the ionic radii and electronegativities of the species present in each site, given by the following expressions (see Methods):

2 $$r_{\mathrm{avg}} = xr_{\mathrm{X}} + \left( {1 - x} \right)r_{\mathrm{Y}}$$

3 $$\chi _{\mathrm{avg}} = \chi _{\mathrm{O}} - \sqrt {x\left( {\chi _{\mathrm{X}} - \chi _{\mathrm{O}}} \right)^2 + \,\left( {1 - x} \right)\left( {\chi _{\mathrm{Y}} - \chi _{\mathrm{O}}} \right)^2}$$

where X and Y are the species present in a site with fraction *x* and (1−*x*), respectively, and O refers to the element oxygen. The implicit assumption in this “averaged” ANN model is that species X and Y are completely disordered, i.e., different orderings of X and Y result in negligible DFT energy differences.

Using the same 6-24-1 ANN architecture, we fitted an “averaged” model using the energy of the ground state ordering of the 635 unmixed and 772 mixed garnets. We find that the training, validation, and test MAEs of the optimized model are 22, 26, and 26 meV atom^−1^, respectively (Supplementary Fig. 3a). These MAEs are about double that of the unmixed ANN model, but still comparable to the error of the DFT *E*_*f*_ relative to experiments. The larger MAEs may be attributed to the fact that the effect of species orderings on the crystal energy is not accounted for in this “averaged” model.

### Ordered neural network model for mixed garnets

In the second approach, we undertook a more ambitious effort to account for the effect of species orderings on crystal energy. Here, we discuss the results for species mixing on the C site only, for which the largest number of computed compositions and orderings is available. For 2:1 mixing, there are 20 symmetrically distinct orderings within the primitive garnet cell, which can be encoded using a 5-bit binary array [*b*_0_,*b*_1_,*b*_2_,*b*_3_,*b*_4_]. This binary encoding scheme is significantly more compact that the commonly used one-hot encoding scheme, and hence, minimizes the increase in the descriptor dimensionality. We may then modify Eq. 1 as follows:

4 $$E_f = f\left( {\chi _{C^\prime },\,r_{C^\prime },\,\chi _{C^{\prime\prime} },\,r_{C^{\prime\prime} },\,\chi _A,\,r_A,\;\chi _D,\,r_D,\,b_0,\,b_1,\,b_2,\,b_3,\,b_4} \right)$$

where the electronegativities and ionic radii of both species on the C sites are explicitly represented. In contrast to the “averaged” model, we now treat the 20 ordering-*E*_*f*_ pairs at each composition as distinct data points. Each unmixed composition was also included as 20 data points with the same descriptor values and *E*_*f*_, but different binary encodings.

We find that a two-hidden-layer DNN is necessary to model this more complex composition-ordering-energy relationship. The final optimized 13-22-8-1 model exhibits overall training, validation and test MAEs of ~11–12 meV atom^−1^ on the entire unmixed and mixed dataset (Supplementary Fig. 3b). The comparable MAEs between this extended DNN model and the unmixed ANN model is clear evidence that the DNN model has successfully captured the additional effect of orderings on *E*_*f*_. We note that the average standard deviation of the predicted *E*_*f*_ of different orderings of unmixed compositions using this extended DNN model is only 2.8 meV atom^−1^, indicating that the DNN has also learned the fact that orderings of the same species on a particular site have little effect on the energy. Finally, similar MAEs can be achieved for A and D site mixing (Supplementary Fig. 3c and 3d) using the same approach.

### Stability classification of garnets using ANN models

**Fig. 4:**
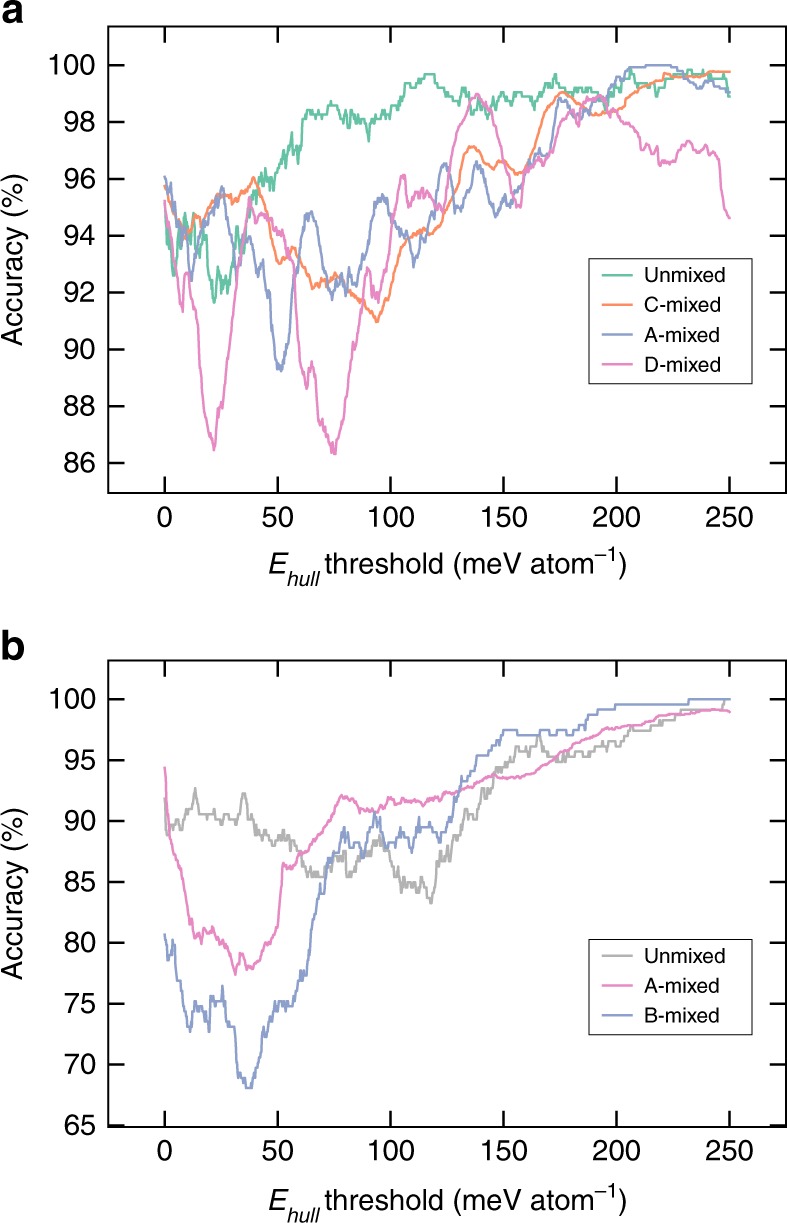
Accuracy of stability classification. Plots of the accuracy of stability classification of the ANN models compared to DFT as a function of the *E*_hull_ threshold for **a**. garnets, and **b**. perovskites. The accuracy is defined as the sum of the true positive and true negative classification rates. A true positive (negative) means that the *E*_hull_ for a particular composition predicted from the optimized artificial neural network model and DFT are both below (above) the threshold. For the mixed compositions, an *E*_hull_ is calculated for all orderings (20, 7, and 18 orderings per composition for C-, A-, and D-mixed garnets, respectively, and ten orderings per composition for both A- and B-mixed perovskites)

While *E*_*f*_ is a good target metric for a predictive ANN model, the stability of a crystal is ultimately characterized by its *E*_hull_. Using the predicted *E*_*f*_ from our DNN models and pre-calculated DFT data from the Materials Project^31^, we have computed *E*_hull_ by constructing the 0 K C-A-D-O phase diagrams. From Fig. 4a, we may observe that the extended C-mixed DNN model can achieve a >90% accuracy in classifying stable/unstable unmixed garnets at a strict *E*_hull_ threshold of 0 meV atom^−1^ and rises rapidly with increasing threshold. Similarly, high classification accuracies of greater than 90% are achieved for all three types of mixed garnets. Given the great flexibility of the garnet prototype in accommodating different species, there are potentially millions of undiscovered compositions. Even using our restrictive protocol of single-site mixing in specified ratios, 8427 mixed garnet compositions can be generated, of which 2307 are predicted to have *E*_hull_ of 0 meV atom^−1^, i.e., potentially synthesizable (Supplementary Fig. 4a). A web application that computes *E*_*f*_ and *E*_hull_ for any garnet composition using the optimized DNNs has been made publicly available for researchers at http://crystals.ai.

### Neural network models for unmixed and mixed perovskites

To demonstrate that our proposed approach is generalizable and not specific to the garnet crystal prototype, we have constructed similar neural network models using a dataset of 240 unmixed, 222 A-mixed and 80 B-mixed ABO_3_ perovskites generated using the species in Supplementary Table 5. We find that a 4-12-1 single-hidden-layer neural network is able to achieve MAEs of 21–34 meV atom^−1^ in the predicted *E*_*f*_ for unmixed perovskites (Fig. 3c), while two 10-24-1 neural networks are able to achieve MAEs of 22–39 meV atom^−1^ in the *E*_*f*_ of the mixed perovskites (Supplementary Fig. 5). These MAEs are far lower than those of prior ML models of unmixed perovskites, which generally have MAEs of close to 100 meV atom^−1^ or higher^9,16^. As shown in Fig. 3b, the accuracy of classifying stable versus unstable perovskites exceeds 80% at a strict *E*_hull_ threshold of 0 meV atom^−1^ and maintains at above 70% at a loosened *E*_hull_ threshold of 30 meV atom^−1^. During the review of this work, a new work by Li et al.^32^ reported achieving comparable MAEs of ~28 meV atom^−1^ in predicting the *E*_hull_ of perovskites using a kernel ridge regression model. However, this performance was achieved using a set of 70 descriptors, with model performance sharply dropping with less than 70 descriptors. Furthermore, Li et al.’s model is restricted to perovskites with *E*_hull_ < 400 meV atom^−1^ and only a single ordering for each mixed perovskite, while in this work, the highest *E*_hull_ is 747 meV atom^−1^ for the perovskite dataset and all symmetrically distinct orderings on the A and B sites within a √2×√2×1 orthorhombic conventional perovskite unit cell (ten structures each) are considered.

## Discussion

To summarize, we have shown that NN models can quantify the relationship between traditionally chemically intuitive descriptors, such as the Pauling electronegativity and ionic radii, and the energy of a given crystal prototype. A key advantage of our proposed NN models is that they rely only on an extremely small number (two) of site-based descriptors, i.e., no structural degrees of freedom are considered beyond the ionic radii of a particular species in a site and the ordering of the cations in the mixed oxides. This is in stark contrast to most machine-learning models in the literature utilizing a large number of correlated descriptors, which render such models highly susceptible to overfitting, or machine-learning force-fields, which can incorporate structural and atomic degrees of freedom but at a significant loss of transferability to different compositions. Most importantly, we derive two alternative approaches—a rigorously defined averaging scheme to model complete cation disorder and a binary encoding scheme to account for the effect of orderings—to extend high-performing unmixed deep learning models to mixed cation crystals with little/no loss in error performance and minimal increase in descriptor dimensionality. It should be noted that our NN models are still restricted to the garnet and perovskite compositions (with or without cation mixing) with no vacancies, though further extensions to other common crystal structure prototypes and to account for vacancies should in principle be possible. Finally, we show how predictive models of *E*_*f*_ can be combined with existing large public databases of DFT computed energies to predict *E*_hull_ and hence, phase stability. These capabilities can be used to efficiently traverse large chemical spaces of unmixed and mixed crystals to identify stable compositions and orderings, greatly accelerating the potential for novel materials discovery.

## Methods

### DFT calculations

All DFT calculations were performed using Vienna ab initio simulation package (VASP) within the projector augmented-wave approach^33,34^. Calculation parameters were chosen to be consistent with those used in the Materials Project, an open database of pre-computed energies for all known inorganic materials^31^. The Perdew-Burke-Ernzehof generalized gradient approximation exchange-correlation functional^35^ and a plane-wave energy cut-off of 520 eV were used. Energies were converged to within 5 × 10^−5^ eV atom^-1^, and all structures were fully relaxed. For mixed compositions, symmetrically distinct orderings within the 80-atom primitive garnet unit cell and the 40-atom √2×√2×1 orthorhombic perovskite supercell were generated using the enumlib library^36^ via the Python Materials Genomics package.^37^

### Training of ANNs

Training of the ANNs was carried out using the Adam optimizer^38^ at a learning rate of 0.2, with the mean square error of *E*_*f*_ as the loss metric. For each architecture, we ran with a random 64:16:20 split of training, validation and test data, i.e., random sub-sampling cross validation.

### Electronegativity averaging

Pauling’s definition of electronegativity is based on an “additional stabilization” of a heteronuclear bond X–O compared to average of X–X and O–O bonds, as follows.

$$\left( {\chi _{\mathrm{X}} - \chi _O} \right)^2 = E_d\left( {{\mathrm{XO}}} \right) - \frac{{E_d\left( {{\mathrm{XX}}} \right) + E_d\left( {{\mathrm{OO}}} \right)}}{2}$$

where *χ*_X_ and *χ*_O_ are the electronegativities of species X and O, respectively, and *E*_*d*_ is the dissociation energy of the bond in parentheses. Here, O refers to oxygen.

For a disordered site containing species X and Y in the fractions *x* and (1−*x*), respectively, we obtain the following:

$$\left( {\chi _{\mathrm{X}_x\mathrm{Y}_{1 - x}} - \chi _O} \right)^{2} 	= xE_d\left( {{\mathrm{XO}}} \right) + \left( {1 - x} \right)E_d\left( {{\mathrm{YO}}} \right) \\ 	- \frac{{xE_d\left( {{\mathrm{XX}}} \right) + \left( {1 - x} \right)E_d\left( {{\mathrm{YY}}} \right) + E_d\left( {{\mathrm{OO}}} \right)}}{2}\\ 	= x\left( {\chi _{\mathrm{X}} - \chi _{\mathrm{O}}} \right)^2 + \left( {1 - x} \right)\left( {\chi _{\mathrm{Y}} - X_{\mathrm{O}}} \right)^2$$

We then obtain the effective electronegativity for the disordered site as follows:

$${\mathrm{\chi }}_{\mathrm{X}_x\mathrm{Y}_{1 - x}} = \chi _{\mathrm{O}} - \sqrt {x\left( {\chi _{\mathrm{X}} - \chi _{\mathrm{O}}} \right)^2 + \left( {1 - x} \right)\left( {\chi _{\mathrm{Y}} - \chi _{\mathrm{O}}} \right)^2}$$

## Electronic supplementary material

Supplementary Information

Peer Review File

## Data Availability

The datasets generated during and/or analysed during the current study are available in the GitHub repository https://github.com/materialsvirtuallab/garnetdnn as well as the Dryad Digital Repository (doi: 10.5061/dryad.760r5b6). A web application that estimates *E*_*f*_ and *E*_hull_ for any given garnet or perovskite composition using the optimized DNNs is available at http://crystals.ai/.
