# Supplementary material for: Deep neural networks for accurate predictions of crystal stability
Source: Nat Commun. 2018 Sep 18;9:3800. doi: 10.1038/s41467-018-06322-x (PMC6143552; doi:10.1038/s41467-018-06322-x)
Supplement: Supplementary file 1 — Supplementary Information [file 41467_2018_6322_MOESM1_ESM.pdf]

Supplementary Information for:

Deep Neural Networks for Accurate Predictions of Crystal Stability

By Weike Ye *et al*.

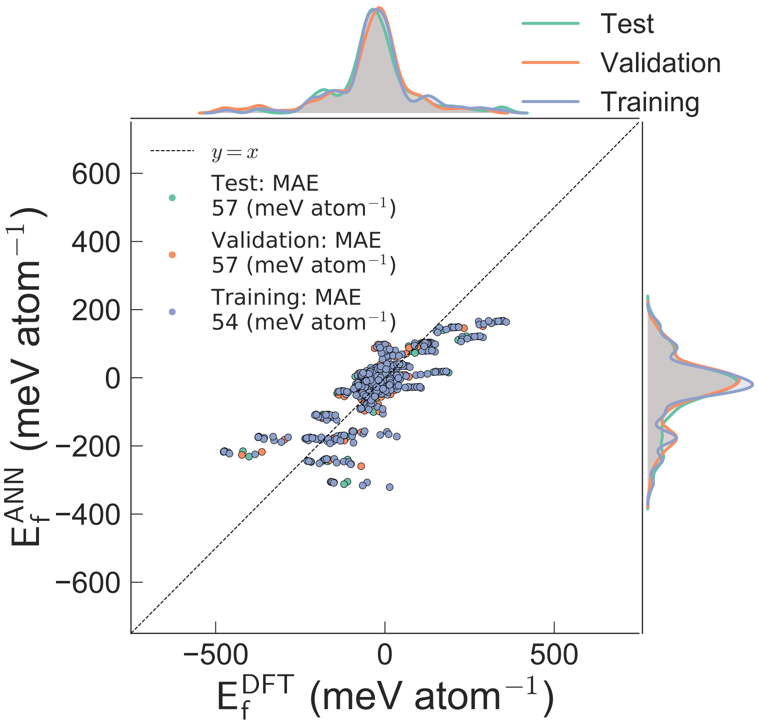

Supplementary Figure 1 | Performance of multiple linear regression model on E_f^{DFT} of unmixed garnets. The high training, validation and test mean absolute errors (MAEs) of 54, 57 and 57 meV atom^{-1} indicate that a simple linear functional form is insufficient to model the relationship between E_f^{DFT} and the Pauling electronegativity and ionic radii descriptors. The R^2 for training, validation and test data are 0.63, 0.63 and 0.63, respectively. The black line (dashed) in the figure is the identity line serving as reference.

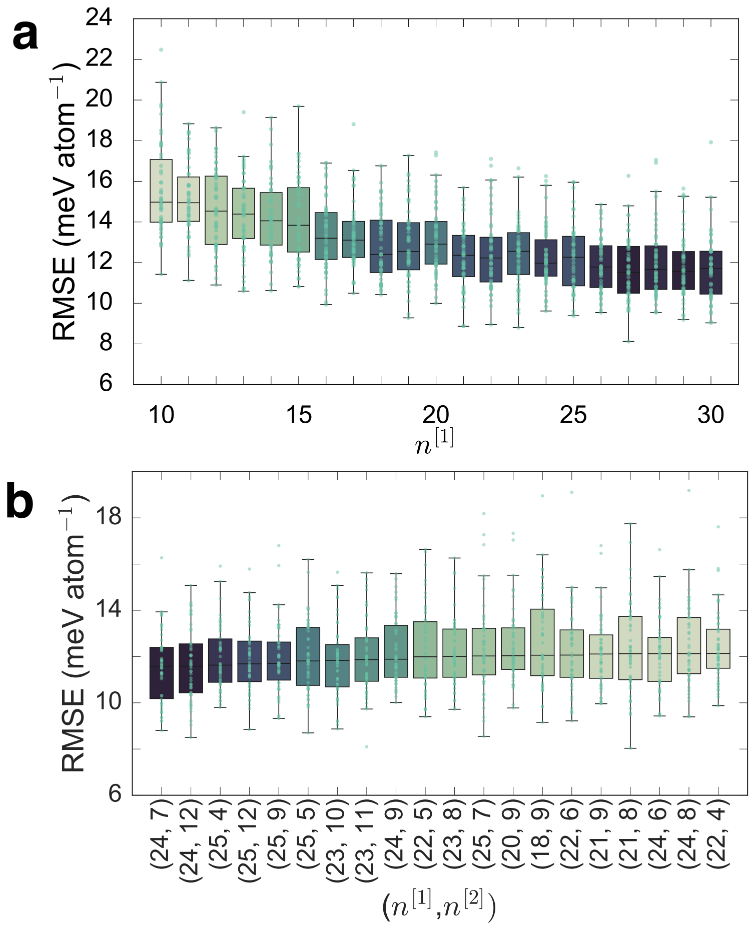

Supplementary Figure 2 | Optimization of artificial neural network (ANN) architecture. a, Plot of the root mean square error (RMSE) loss metric versus number of neurons in a single-hidden-layer ANN model. The RMSE converges at $n^{[1]}\sim 20$, and the smallest standard deviation is observed at $n^{[1]} = 24$. **b,** Plot of the RMSE loss metric versus number of neurons in a two-hidden-layer deep neural network (DNN) model for unmixed garnets. Only the 20 best-performing models are shown for brevity. The RMSE loss metric achieved by the DNN model is similar to that of the single-hidden-layer ANN model. The box shows the quartiles of the dataset while the whiskers extend to show the rest of the distribution.

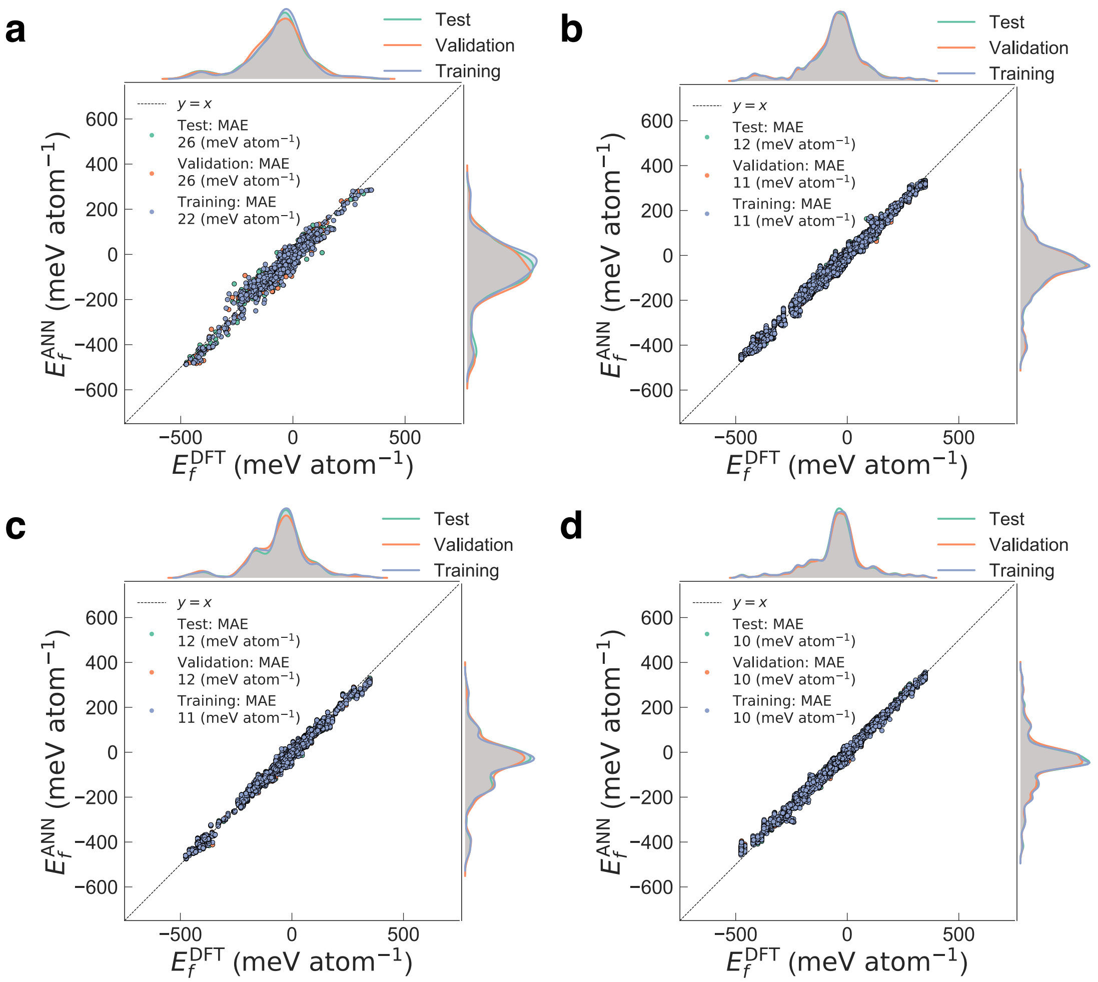

Supplementary Figure 3 | Performance of optimized artificial neural network models for garnets. Plot of E_f^{NN} against E_f^{DFT} for **a.** “averaged” ANN model trained on all unmixed and mixed garnets, **b.** ordered DNN model trained on unmixed garnets with C-mixed garnets(standard deviations of E_f^{DFT} for training, validation and test set are: 130, 128 and 130 meV atom^{-1}), **c.** ordered DNN model trained on unmixed garnets with A-mixed garnets (standard deviations of E_f^{DFT} for training, validation and test set are: 132, 134 and 131 meV atom^{-1}), and **d.** ordered DNN model trained on unmixed garnets with D-mixed garnets (standard deviations of E_f^{DFT} for training, validation and test set are: 126, 126 and 127 meV atom^{-1}). The black lines (dashed) in all subfigures are the identity lines serving as references.

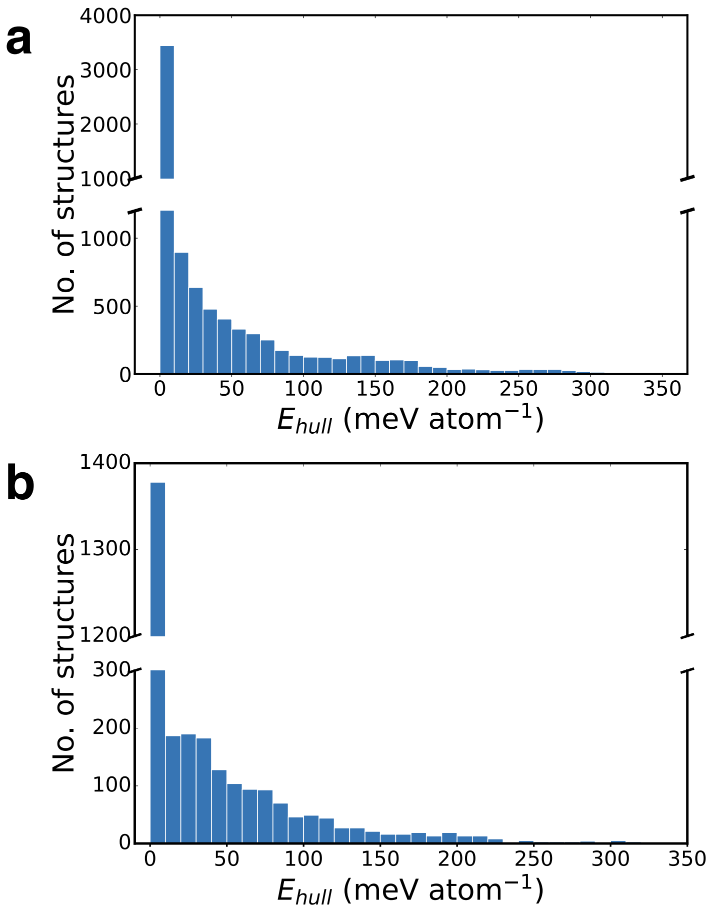

Supplementary Figure 4 | Histograms of E_{hull} predicted using the optimized neural network models for garnets and perovskites. a. A total of 8,427 garnet compositions were generated based on 2:1 mixing on the C or D sites, or 1:1 mixing on the A site. Only the ordering with the lowest E_{hull} is presented at each composition. Of the 8,385 compositions, 2,307 compositions are predicted to have E_{hull} = 0 meV atom^{-1}. **b.** A total of 2,791 perovskite compositions were generated based on 1:1 mixing on the A or D sites. Only the ordering with lowest E_{hull} is presented at each composition. Of the 2,791 compositions, 1,147 compositions are predicted to have E_{hull} = 0 meV atom^{-1}.

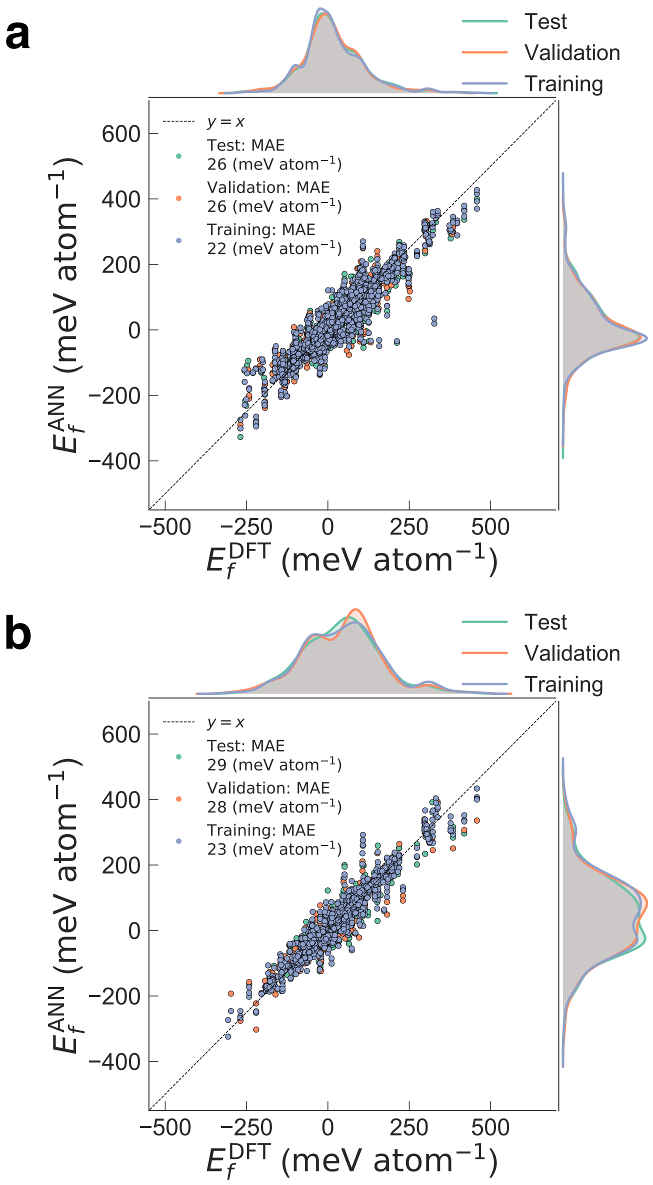

Supplementary Figure 5 | Performance of optimized artificial neural network models for perovskites. Plot of E_f^{ANN} against E_f^{DFT} for **a.** ordered ANN model trained on unmixed with A-mixed perovskites (standard deviation of E_f^{DFT} for training, validation and test sets are: 95, 94 and 96 meV atom^{-1}), and **b.** ordered ANN model trained on unmixed with B-mixed perovskites (standard deviations of E_f^{DFT} for training, validation and test sets are: 121, 117 and 115 meV atom^{-1}). The black lines (dashed) in a. and b. are the identity lines serving as references.

Supplementary Table 1 | Binary oxides used as reference states used for garnet E_f calculations.

Element	Oxidation State	Binary Oxide	ICSD ID	Materials ID
Na	1	Na_2O	60435	mp-2352
Li	1	Li_2O	57411	mp-1960
Ag	1	Ag_2O	174087	mp-353
Cs	1	Cs_2O	27919	mp-7988
Rb	1	Rb_2O	77676	mp-1394
K	1	K_2O	180571	mp-971
Tl	1	Tl_2O	77699	mp-27484
Cd	2	CdO	24802	mp-1132
Zn	2	ZnO	31060	mp-2133
Ba	2	BaO	52278	mp-1342
Sr	2	SrO	28904	mp-2472
Ca	2	CaO	52783	mp-2605
Mg	2	MgO	52026	mp-1265
Be	2	BeO	29271	mp-2542
Co	2	CoO	53057	mp-19079
Ni	2	NiO	60435	mp-19009
Hg	2	HgO	40316	mp-1224
Pb	2	PbO	26596	mp-672237
Pd	2	PdO	26598	mp-1336

Continued from previous page

Element	Oxidation State	Binary Oxide	ICSD ID	Materials ID
Cu	2	CuO	628618	mp-1692
La	3	La_2O_3	96201	mp-2292
Cr	3	Cr_2O_3	201102	mp-19399
Al	3	Al_2O_3	43732	mp-1143
Bi	3	Bi_2O_3	94229	mp-23262
Pr	3	Pr_2O_3	96203	mp-16705
Sm	3	Sm_2O_3	647461	mp-218
Eu	3	Eu_2O_3	40472	[1]
Gd	3	Gd_2O_3	33652	mp-504886
Tb	3	Tb_2O_3	647509	mp-1056
Dy	3	Dy_2O_3	27994	mp-2345
Ho	3	Ho_2O_3	41268	mp-812
Er	3	Er_2O_3	630897	mp-679
Tm	3	Tm_2O_3	78582	mp-1767
Yb	3	Yb_2O_3	33658	mp-2814
Lu	3	Lu_2O_3	151762	mp-1427
Y	3	Y_2O_3	181825	mp-2652

[1] There is no corresponding entry in MP. The energy was obtained by applying DFT calculation on the structure using MP-compatible parameters.

Continued from previous page

Element	Oxidation State	Binary Oxide	ICSD ID	Materials ID
Rh	3	Rh_2O_3	181829	mp-542734
Ga	3	Ga_2O_3	166198	mp-886
Sc	3	Sc_2O_3	647397	mp-216
Nd	3	Nd_2O_3	645664	mp-1045
Au	3	Au_2O_3	8014	mp-27253
B	3	B_2O_3	36066	mp-306
Mn	3	Mn_2O_3	76087	mp-542877
Hf	4	HfO_2	27313	mp-352
Zr	4	ZrO_2	68782	mp-2858
Ge	4	GeO_2	92551	mp-470
Ti	4	TiO_2	69331	mp-2657
Si	4	SiO_2	200726	mp-7000
Ru	4	RuO_2	56007	mp-825
Sn	4	SnO_2	39173	mp-856
Pt	4	PtO_2	647320	mp-1285
Mo	4	MoO_2	36263	mp-510536
Re	4	ReO_2	24060	mp-7228

Continued from previous page

Element	Oxidation State	Binary Oxide	ICSD ID	Materials ID
Se	4	SeO_2	412234	mp-726
Te	4	TeO_2	26844	mp-2125
In	3	In_2O_3	181833	mp-22598
Tc	4	TcO_2	173153	mp-33137
Ir	4	IrO_2	640887	mp-2723
Os	4	OsO_2	30400	mp-996
Nb	5	Nb_2O_5	25750	[2]
P	5	P_2O_5	40865	mp-562613
Sb	5	Sb_2O_5	1422	mp-1705
Ta	5	Ta_2O_5	[3]	mvc-4415
As	5	As_2O_5	10015	mp-555434
V	5	V_2O_5	40488	mp-25620
W	6	WO_3	50728	mp-19342
Fe	3	α-Fe_2O_3	161283	mp-24972
Fe	2	FeO	633029	mp-18905

[2] There is no corresponding entry in MP. The energy was obtained by applying DFT calculation on the structure using MP-compatible parameters.

[3] This structure is not included in ICSD, but the DFT calculation from MP shows that it has a calculated formation energy of -23.489 eV formula unit(fu)$^{-1}$, which is close to reported experimental value (-21.209 eV fu^{-1})[4].

Supplementary Table 2 | Elements used in generating perovskite and corresponding binary oxides and used as reference states used for perovskite E_f calculations.

Element	Oxidation State	Binary Oxide	ICSD ID	Materials ID
Ag	1	Ag_2O	173984	mp-353
Al	3	Al_2O_3	60419	mp-1143
Au	3	Au_2O_3	8014	mp-27253
As	5	As_2O_5	10015	mp-555434
Ba	2	BaO	616004	mp-1342
Bi	3	Bi_2O_3	15072	mp-23262
Ca	2	CaO	60704	mp-2605
Cd	2	CdO	181057	mp-1132
Ce	3	Ce_2O_3	96202	mp-542313
Ce	4	CeO_2	164225	mp-20194
Co	2	CoO	9865	mp-19079
Co	3	Co_2O_3		mvc-852
Cr	3	Cr_2O_3	201102	mp-19399
Cr	4	CrO_2	166021	mp-19177
Cs	1	Cs_2O	27919	mp-7988
Cu	2	CuO	653723	mp-1692
Dy	3	Dy_2O_3	96208	mp-2345
Er	3	Er_2O_3	39521	mp-679
Eu	3	Eu_2O_3	40472	
Fe	2	FeO	633029	mp-18905
Fe	3	Fe_2O_3	161283	mp-24972
Fe	4	FeO_2		mp-850222
Ga	3	Ga_2O_3	34243	mp-886
Gd	3	Gd_2O_3	152449	mp-504886
Ge	4	GeO_2	158592	mp-470
Hf	4	HfO_2	172165	mp-352

Continued from previous page

Element	Oxidation State	Binary Oxide	ICSD ID	Materials ID
Hg	2	HgO	40316	mp-1224
Ho	3	Ho_2O_3	44516	mp-812
I	5	I_2O5	182672	mp-23261
In	3	In_2O_3	640179	mp-22598
Ir	4	IrO_2	84577	mp-2723
K	1	K_2O	44674	mp-971
La	3	La_2O_3	96201	mp-2292
Li	1	Li_2O	54368	mp-1960
Lu	3	Lu_2O_3	642477	mp-1427
Mg	2	MgO	41990	mp-1265
Mn	2	MnO	28898	mp-714882
Mn	3	Mn_2O_3	9091	mp-542877
Mn	4	MnO_2	20227	mp-19395
Mo	4	MoO_2	99714	mp-510536
Na	1	Na_2O	180570	mp-2352
Nb	4	NbO_2	35181	mp-557057
Nb	5	Nb_2O_5	25750	
Nd	3	Nd_2O_3	645664	mp-1045
Ni	2	NiO	61318	mp-19009
Os	4	OsO_2	30400	mp-996
P	5	P_2O_5	40865	mp-562613
Pb	2	PbO	99777	mp-672237
Pb	4	PbO_2	43460	mp-20725
Pd	4	PdO_2	647283	mp-1018886
Pd	2	PdO	29281	mp-1336
Pr	3	Pr_2O_3	96203	mp-16705

Continued from previous page

Element	Oxidation State	Binary Oxide	ICSD ID	Materials ID
Pr	4	PrO_2	647300	mp-1302
Pt	2	PtO	164290	mp-7947
Pt	4	PtO_2	647320	mp-1285
Pu	4	PuO_2	55456	mp-1959
Rb	1	Rb2O	180572	mp-1394
Re	4	ReO2	24060	mp-7228
Rh	3	Rh2O3	108941	mp-542734
Sc	3	Sc2O3	647397	mp-216
Se	4	SeO2	59712	mp-726
Si	4	SiO2	200726	mp-7000
Sm	3	Sm2O3	647461	mp-218
Sn	4	SnO2	39173	mp-856
Sr	2	SrO	180194	mp-2472
Tc	4	TcO2	173152	mp-33137
Ta	5	Ta2O5	-	mvc-4415
Tb	3	Tb2O3	40474	mp-1056
Tb	4	TbO2	647500	mp-2458
Te	4	TeO2	26844	mp-2125
Ti	3	Ti2O3	77696	mp-458
Ti	4	TiO2	202240	mp-2657
Tl	1	Tl2O	16220	mp-27484
Tl	3	Tl2O3	74090	mp-1658
Tm	3	Tm2O3	647581	mp-1767
V	3	V2O3	260212	mp-25787
V	4	VO2	1504	mp-19094
V	5	V2O5	99808	mp-25620
W	4	WO2	8217	mp-19372

Continued from previous page

Element	Oxidation State	Binary Oxide	ICSD ID	Materials ID
Y	4	Y2O3	23811	mp-2652
Yb	3	Yb2O3	62872	mp-2814
Zn	2	ZnO	647681	mp-2133
Zr	4	ZrO2	172161	mp-2858

Supplementary Table 3 | Species on the C, A and D sites in garnet, adapted from ref. [1]

Site	Ions
A	Li^{+}, Dy^{3+}, Y^{3+}, Ho^{3+}, Er^{3+}, Tm^{3+}, Lu^{3+}, Hf^{4+}, Mg^{2+}, Zr^{4+}, Sc^{3+}, Ta^{5+}, Ti^{4+}, Nb^{5+}, Al^{3+}, Zn^{2+}, Cr^{3+}, In^{3+}, Ga^{3+}, Sn^{4+}, Ge^{4+}, Sb^{5+}, Ru^{4+}, Rh^{3+}
C	Ba^{2+}, Na^{+}, Sr^{2+}, Ca^{2+}, Tb^{3+}, La^{3+}, Pr^{3+}, Nd^{3+}, Sm^{3+}, Gd^{3+}, Eu^{3+}, Dy^{3+}, Y^{3+}, Ho^{3+}, Er^{3+}, Tm^{3+}, Lu^{3+}, Hf^{4+}, Mg^{2+}, Zr^{4+}, Zn^{2+}, Cd^{2+}, Bi^{3+}
D	Li^{+}, Ti^{4+}, Al^{3+}, Ga^{3+}, Si^{4+}, Sn^{4+}, Ge^{4+}, As^{5+}, P^{5+}

Supplementary Table 4 | Accuracy of DFT formation energies versus experiments.

Formula	E_f^{EXP} (meV atom^{-1})	E_f^{DFT} (meV atom^{-1})	Source
$Dy_3Al_5O_{12}$	-51[1]	-54	Ref. [2]
$Ho_3Al_5O_{12}$	-53[1]	-51	Ref. [2]
$Er_3Al_5O_{12}$	-50[1]	-49	Ref. [2]
$Tm_3Al_5O_{12}$	-50[1]	-46	Ref. [2]
$Lu_3Al_5O_{12}$	-38[1]	-37	Ref. [2]
$Y_3Al_5O_{12}$	-60[1]	-51	Ref. [2]
$Sm_3Ga_5O_{12}$	-76[1]	-67	Ref. [2]
$Eu_3Ga_5O_{12}$	-72[1]	-28	Ref. [2]
$Gd_3Ga_5O_{12}$	-76[1]	-53	Ref. [2]
$Dy_3Ga_5O_{12}$	-62[1]	-53	Ref. [2]
$Ho_3Ga_5O_{12}$	-66[1]	-48	Ref. [2]
$Er_3Ga_5O_{12}$	-62[1]	-44	Ref. [2]
$Tm_3Ga_5O_{12}$	-56[1]	-38	Ref. [2]
$Lu_3Ga_5O_{12}$	-45[1]	-25	Ref. [2]
$Y_3Ga_5O_{12}$	-69[1]	-52	Ref. [2]
$Ca_3Al_2Si_3O_{12}$	-169	-132	Ref. [3]

[1] Measured at 977 K.

E_f^{EXP} is the enthalpy of formation of garnets from binary oxides, i.e., the enthalpy change of the reaction 3/2 Ln_2O_3 + 5/2 M_2O_3 $\rightarrow$ $Ln_3M_5O_{12}$ (Ln = Rare Earth, M=Al, Ga), and E_f^{DFT} is the DFT computed formation energy based on the same reaction. The mean absolute error (MAE) between E_f^{EXP} and E_f^{DFT} is ~ 14 meV $atom^{-1}$.

Supplementary Table 5 | Species on the A and B sites in perovskites

Site	Ions
A	Ba^{2+}, Sr^{2+}, Ca^{2+}, La^{3+}, Tb^{3+}, Ce^{3+}, Ce^{4+}, Pr^{3+}, Nd^{3+}, Sm^{3+}, Gd^{3+}, Dy^{3+}, Y^{3+}, Ho^{3+}, Er^{3+}, Tm^{3+}, Mg^{2+}, Sc^{3+}, Mn^{2+}, Al^{3+}, Tl^{3+}, Zn^{2+}, Cd^{2+}, Ni^{2+}, Sn^{4+}, Bi^{3+}, Pd^{2+}, Pt^{2+}, Rh^{3+}, Pb^{2+}
B	La^{3+}, Tb^{3+}, Ce^{3+}, Ce^{4+}, Pr^{3+}, Nd^{3+}, Sm^{3+}, Eu^{3+}, Gd^{3+}, Dy^{3+}, Y^{3+}, Ho^{3+}, Er^{3+}, Tm^{3+}, Lu^{3+}, Hf^{4+}, Mg^{2+}, Zr^{4+}, Sc^{3+}, Ta^{5+}, Ti^{4+}, Mn^{2+}, Mn^{4+}, Al^{3+}, Tl^{3+}, V^{5+}, Zn^{2+}, Cr^{3+}, In^{3+}, Ga^{3+}, Fe^{2+}, Fe^{3+}, Co^{2+}, Co^{3+}, Cu^{2+}, Re^{4+}, Si^{4+}, Tc^{4+}, Ni^{2+}, Sn^{4+}, Ge^{4+}, Bi^{3+}, Mo^{4+}, Ir^{4+}, Os^{4+}, Pd^{4+}, Ru^{4+}, Pt^{4+}, Rh^{3+}, Pb^{4+}, W^{4+}, Au^{3+}

Supplementary References

1. Granat-struktur, D., Ubersicht, E., Kationen, D. & Ionenverteilung, D. Crystal Chemistry of the Garnet. *Zeitschrift für Krist. - Cryst. Mater.* **47,** 1989 (1999).
2. Kanke, Y. & Navrotsky, A. A Calorimetric Study of the Lanthanide Aluminum Oxides and the Lanthanide Gallium Oxides: Stability of the Perovskites and the Garnets. *J. Solid State Chem.* **141,** 424–436 (1998).
3. Cemič, L. *Thermodynamics in mineral sciences: An introduction*. *Thermodynamics in Mineral Sciences: An Introduction* (Springer-Verlag, 2005).
4. Kubaschewski, O., Alcock, C. B. & Spencer, P. J. *Materials thermochemistry*. (Pergamon Press, 1993).
