# Supplementary material for: Deep neural networks for accurate predictions of crystal stability
Source: Nat Commun. 2018 Sep 18;9:3800. doi: 10.1038/s41467-018-06322-x (PMC6143552; doi:10.1038/s41467-018-06322-x)
Supplement: Supplementary file 2 — Peer Review File [file 41467_2018_6322_MOESM2_ESM.pdf]

Editorial Note: This manuscript has been previously reviewed at another journal that is not operating a transparent peer review scheme. The manuscript was considered suitable for publication without further review at *Nature Communications*.
